# A dataset of synthetic art dialogues with ChatGPT

**DOI:** 10.1038/s41597-024-03661-x

**Published:** 2024-07-27

**Authors:** Manuel Gil-Martín, Cristina Luna-Jiménez, Sergio Esteban-Romero, Marcos Estecha-Garitagoitia, Fernando Fernández-Martínez, Luis Fernando D’Haro

**Affiliations:** Speech Technology and Machine Learning Group (T.H.A.U. Group), Information Processing and Telecommunications Center, E.T.S.I. de Telecomunicación, Universidad Politécnica de Madrid, 28040 Madrid, Spain

**Keywords:** Databases, Engineering

## Abstract

This paper introduces Art_GenEvalGPT, a novel dataset of synthetic dialogues centered on art generated through ChatGPT. Unlike existing datasets focused on conventional art-related tasks, Art_GenEvalGPT delves into nuanced conversations about art, encompassing a wide variety of artworks, artists, and genres, and incorporating emotional interventions, integrating speakers’ subjective opinions and different roles for the conversational agents (e.g., teacher-student, expert guide, anthropic behavior or handling toxic users). Generation and evaluation stages of GenEvalGPT platform are used to create the dataset, which includes 13,870 synthetic dialogues, covering 799 distinct artworks, 378 different artists, and 26 art styles. Automatic and manual assessment proof the high quality of the synthetic dialogues generated. For the profile recovery, promising lexical and semantic metrics for objective and factual attributes are offered. For subjective attributes, the evaluation for detecting emotions or subjectivity in the interventions achieves 92% of accuracy using LLM-self assessment metrics.

## Background & Summary

The recent arrival of powerful generative Large Language Models (LLMs) has increased the interest in natural language processing, presenting unprecedented opportunities for the creation of synthetic dialogues that emulate human characteristics. These models, equipped with advanced capabilities for understanding and generating text, have become a potent tool in the development of datasets specifically designed to explore the intricacies of human-like conversations. This capacity could be mixed with the information of other datasets not focused on dialogues that include a wide variety of information related to a specific topic, such as bank marketing, housing stock or art.

In the expansive landscape of available datasets, there exists a rich repository covering diverse domains, such as gesture or emotion recognition, toxicity evaluation, general dialogues or specific topic domains like art. This section describes existing datasets focused on dialogues or art information.

For instance, MultiWOZ^1^ is a multi-domain dataset for task-oriented dialogues modelling which includes 8,438 dialogues with average 13.68 turns per dialogue related to different domains such as restaurant, attraction, hotel, taxi, train, hospital or police. Another example is the Topical-Chat dataset^2^, a large collection of human-human knowledge-grounded open-domain conversations that consists of 11,319 dialogs and 248,014 utterances. This dataset contains 8 broad topics (fashion, politics, books, sports, general entertainment, music, science & technology and movies) and conversation partners without explicitly defined roles.

Other datasets are the PersonaChat dataset^3^, a corpus of human-human persona-conditioned conversations that consists of 10,907 dialogs and 162,064 utterances where each worker is asked to condition their responses on a persona, or the DailyDialog^4^, an English dialog dataset which contains 13,118 dialogues with 8 speaker turns on average. This last dataset was manually labeled with communication intention and emotion information. It contains various communication topics such as ordinary life, relationship, work or tourism and emotions such as anger, disgust, fear, joy, sadness and surprise.

In addition, the DialogCC^5^ dataset contains around 93k diverse real-world dialogues on different topics around several images per dialogue (651k in total). The dialogues were created through a CLIP-based automatic pipeline using meaningful textual and visual features.

Datasets containing dialogues offer valuable insights into conversational dynamics, while datasets dedicated to a specific topic usually provide a great amount of metadata. Regarding the datasets not focused on dialogue, in the case of art, ArtEmis dataset^6^ provides information about various artworks and artists, including details about artistic genres or movements. This dataset is intended to provide a detailed understanding of the interplay between visual content and its emotional effect on people, as well as natural language explanations for the emotional choice. The dataset consists of 455 K emotion attributions and explanations from humans, on around 80 K artworks, including more than 25 painting styles from more than 1000 painters. In addition, other previous works^7,8^ provided valuable question answering datasets related to cultural heritage and art. For example, VISCOUNTH^7^ offers 6.5 M question-and-answer pairs using visual content and an associated natural language description. While the dataset boasts a wealth of cultural heritage information that could greatly benefit visitors to museums and cultural sites, it notably lacks dialogues.

Other previous works^9,10^ focused on generating descriptions of artworks in order to describe multiple aspects of the image such as its style, content, or composition, and provide background and contextual knowledge about the artist, their influences, or the historical period. The generated description could be useful to contextualize the dialogue generation tools.

As described in this section, existing datasets do not integrate both dialogues and comprehensive information about a topic and specific speakers’ behaviors. Specifically, there is a distinct scarcity of datasets that include detailed information about artworks and subjective interventions from the speakers including emotional responses, personal opinions or toxic comments engaging with the artworks. This way, the main contributions of this paper are the following:

- Creating a dataset of dialogues about art including a great variability of artworks, artists and genres. This dataset will be relevant to train or fine-tune conversational models on the specific topic of art, with high factuality, providing instruction-based responses and handling different types of situations in the context of a museum. This is the first time, up to the best of our knowledge, that such a dataset is created and released for the research community.
- Incorporating emotional interventions in art dialogues involving the specific artwork. In this case, the generated dialogues include up to 8 different emotions that can be aroused in the visitors when looking at the given artwork. This characteristic will open the possibility of having emotional-aware systems that can be also complemented with multimodal information for the automatic detection of the emotion in the visitors.
- Integrating speakers’ subjective opinions in art dialogues connecting to the specific artwork. Through the incorporation of subjectivity in the dialogues, the dataset will allow the development of conversational systems that can manifest or not such characteristics, while also handling improper behaviors.

This dataset was created using dialogues automatically generated by using ChatGPT^11^ through prompting engineering techniques. The instructions given to ChatGPT requested the generation of dialogues with specific metadata and speakers’ characteristics. These instructions aimed to provide dialogues with objective information about artworks, convey emotional information about each artwork, and exhibit anthropic behaviors by expressing or withholding opinions and emotions. In addition, the prompt included accurate information about each artwork to reduce the possibility of hallucinations. Finally, we also tested some capabilities of ChatGPT to handle toxic and non-toxic users or sensitive artworks. Such a dataset would empower researchers to create models that not only comprehend the art dialogues but also focus on the human emotions or opinions manifested during the conversation, as well as controlling the behavior of the conversational agent.

The creation of this novel dataset focused on dialogues about art marks a significant milestone in the field. This dataset stands out for its comprehensive coverage, encompassing a diverse range of artworks, artists, and genres. This rich variety ensures that the dataset is well-suited for training and fine-tuning conversational models with a specific emphasis on the domain of art. The dataset contextualization within the art domain, specifically within a museum setting, enhances its real-world applicability. It mimics interactions between an expert chatbot and a user, showcasing different behavioral profiles and conversational goals. This way, the chatbot can mimic anthropic behavior by expressing personal opinions or emotions. Whether the chatbot assumes the role of an intelligent tutor imparting knowledge or adopts a descriptive and informative stance, the dataset provides a versatile foundation for training conversational models. The inclusion of toxicity management ensures that the chatbot can handle various user behaviors in a constructive manner.

This paper is organized as follows. Section 2 describes the material and methods used, including the source of art information, the procedure to select the artworks in the ArtEmis dataset^6,12^, and a description of the system used to generate and evaluate the dialogues of the new Art_GenEvalGPT dataset. Section 3 deeply describes the data records and Section 4 includes the technical validation performed for evaluating the robustness of the generated dataset. Finally, Section 5 summarizes the main conclusions of the paper.

## Material and Methods

This section includes information about the source of information used for retrieving the art metadata, the procedure for selecting the artworks for the dataset and the system architecture for creating the dialogues.

### Source of art information

The ArtEmis dataset^6^ is a rich collection comprising 455 K emotion attributions and explanations, all of which are associated with 80 K artworks sourced from the WikiArt website^13^. The primary objective of this art dataset is to delve into the realm of linguistic affective explanations grounded in visual stimuli. Annotators in this dataset were tasked with explaining and establishing connections between visual attributes within artworks and the corresponding psychological interpretations of emotions.

Within the ArtEmis dataset, annotators operate within a well-defined set of eight categorical emotion states. These emotions include four negative categories: anger, disgust, fear, and sadness, as well as four positive categories: amusement, awe, contentment, and excitement. This categorization framework helps to structure and standardize the emotional attributions provided by the annotators.

**Fig. 1:**
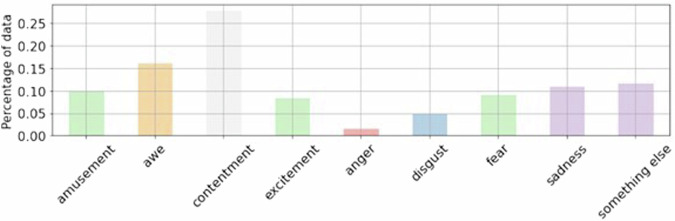
Histogram of emotions captured in ArtEmis. Positive emotions occur significantly more often than negative emotions (62.0% of all responses vs. 26.3%). The annotators use a non-listed emotion (“something-else” category) 11.7% of the times^6^.

The annotation process for ArtEmis was meticulous. For each artwork, a minimum of five annotators were involved. Their task began with expressing their dominant emotional reaction to the artwork, which they did by selecting one of the predefined eight emotion categories or a ninth option labeled “something-else”. This ninth option allowed annotators to express emotions that may not be explicitly listed or to clarify if they did not experience a strong emotional reaction. Figure 1 shows a histogram of the emotions captured in the original ArtEmis dataset. As we can see, the distribution of emotions is not balanced creating an undesired bias.

Following this initial step, annotators were further required to provide detailed explanations in free text. These explanations were expected to contain specific references to the visual elements present within the artwork.

The dataset statistics emphasize its scale and significance. It encompasses a total of 454,684 explanatory utterances and emotional responses, featuring 37,250 distinct words. ArtEmis dataset comprised 80,031 unique artworks created by 1,119 artists. This underlying dataset covers a broad spectrum of 27 art styles (including abstract, baroque, cubism, and impressionism) and 45 genres (including cityscape, landscape, portrait, and still life), thus offering a diverse array of visual stimuli for analysis.

### Selection of artworks from the ArtEmis dataset

To select the artworks included in the proposed Art_GenEvalGPT the dataset, we used the metadata information provided with the first version of ArtEmis dataset^6^, which included not only information about the artwork itself (title, artist, style, etc.), but also detailed understanding of the interplay between visual content, its emotional effect and natural language explanations for the emotion choice among the different annotators.

First of all, although the original total number of reported artists in the ArtEmis dataset article^6^ was 1,119, we filtered 9 artists that were tagged as ‘unknown’ artists, those associated with artworks having problematic coding formats on their names or artwork title, therefore obtaining a total of 1,110 artists, and resulting finally in 799 different artworks in total. Then, we categorized the 1,110 artists as female or male, resulting in 9.27% (103) females and 90.73% (1,007) males. This result highlights the non-balance gender representation of artists in the original dataset. The annotated file with the names of authors and their genders can be downloaded from: https://shorturl.at/tPU15.

As commented in the previous section, the ArtEmis dataset includes multiple dimensions that could be used as a target for balancing our new filtered dataset (i.e., with the goal of reducing biases). Initially, we started considering different dimensions and criteria like balancing the selected dataset by style, artist, gender, year, etc. However, we found it very difficult to balance their distribution following any of these criteria, creating or maintaining undesired biases. Finally, we opted that paintings were ordered considering the consensus or agreement between the human annotators regarding the triggered emotion and balancing the number of selected artworks per emotion. Besides, to avoid noise in the selection process, for each emotion we selected those artworks with a higher inter-annotator agreement (i.e., three or more annotators agreed on the same triggered emotion).

By following this criterion, we also found that it allowed us to balance the distribution of the data of the generated synthetic dataset when considering different emotions, which we consider an important aspect to create an emotion-balanced dialogues dataset.

Therefore, finally we selected 100 artworks for each emotion, resulting in a total of 800 artworks. Table 1 provides some statistics of the final distribution of the Art_GenEvalGPT dataset. As it will be mentioned in the following section, the procedure used for generating and evaluating the dialogues could not be completed for several artworks due to its content filter restrictions or recurring errors during the generation and evaluation processes. Nevertheless, the final dataset comprises 13,870 dialogues, and as we can see, the highest number of artworks do not trigger any emotion (i.e., neutral), but for those that they do, there is a balanced distribution of emotions. The amount of neutral dialogues is higher because for each dialogue, a neutral version was generated.

**Table 1:** Statistics and final distribution of emotions of the Art_GenEvalGPT dataset.

Description	Amount	
Total number of generated synthetic dialogues	13,870	
Total number of different artworks	799	
Total number of different artists	378	
To**t**al number of different art styles	26	
**Distribution of dialogues per emotion**	**Amount**	**%**
Amusement	997	7.2
Anger	745	5.4
Awe	943	6.8
Contentment	936	6.7
Disgust	890	6.4
Excitement	885	6.4
Fear	958	6.9
Neutral	6,378	46.0
Sadness	948	6.8
Something else	190	1.4

### System for dialogues generation and evaluation

To create this dataset, we used a flexible framework called GenEvalGPT^14^. This multi-stage framework generates guided and synthetic dialogues between a human and a ‘personalized’ chatbot following a recipe structure with minimal human intervention. This platform determines the successful creation of the dialogues based on the provided specifications; and evaluates various aspects of emotional and subjective responses. The platform includes a two-path automatic evaluation methodology employing LLM-Self-Assessment evaluations and traditional metrics (which can involve rule-based metrics, formula-based metrics and metrics reported by pre-trained models different to LLMs) to address the evaluation of the generated characteristics and contextual information by the LLM against the data requested in the prompt.

Once the dialogues were created based on specific profiles, GenEvalGPT platform implements several strategies to automatically evaluate the quality of the generated dialogues. One of those strategies is to extract from the dialogues the profile used for creating them, and compare both the extracted and original profiles. Then, the efficacy of this profile reconstruction is subsequently evaluated in a second stage using tailored metrics related to both lexical and semantic aspects. These metrics were Jaccard Index^15^, Accuracy, Levenshtein Distance, WER^16^, BLEU-1^17^, and a cosine similarity metric. This way, it is possible to check if the generated dialogues followed the requested instructions and included the provided metadata.

Concerning the emotional and subjective responses, we coined the term ‘Anthropic’ as the characteristic of expressing emotions OR giving personal opinions, preferences, or subjective judgments. This way, the GenEvalGPT platform uses both LLM-Self-Assessment metrics and tailored metrics to determine if the chatbot was anthropic. To generate these automatic metrics, the model can classify the interventions of a speaker with True or False depending on if they show emotional load or subjectivity. For the tailored metrics, we used Valence Aware Dictionary for sEntiment Reasoning (VADER) metric^18^ for the sentiment analysis and a subjectivity score extracted from the TextBlob library^19^ to report valuable information about the appearance of emotional or subjective aspects in the speakers’ interventions.

For toxic behavior, the interventions of each speaker were evaluated with two tools in order to extract a toxicity score: Azure Content Moderator API^20^ and Detoxify library^21^. The first one offers scores for different categories, which are related to sexually explicit or adult, sexually suggestive or mature or offensive. The second one can detect multiple types of toxic comments such as severe_toxicity, obscene, threat, insult, identity_attack or sexual_explicit, and also consider different identity, religion, racial or disability attributes.

## Data Records

This section describes the Art_GenEvalGPT dataset content regarding generation and evaluation details. The database is available at e-cienciaDatos repository^22^.

The dataset includes conversations between an expert chatbot and a user about art, each one showing different types of behaviors or characteristics, containing a wide variability of artworks, artists and art styles, anthropic and non-anthropic expert’s nature, toxic and non-toxic user’s behavior and two possible conversational goals. The chatbot could assume the role of an intelligent tutor, imparting knowledge about artworks and testing the user’s understanding (i.e., “ToD” or task-oriented domain). Alternatively, it could adopt a descriptive and informative stance, offering in-depth insights into artworks. The user’s role complements these goals: as a student, the user interacts by asking questions, providing correct or incorrect answers (which the chatbot could provide feedback), and seeking emotional responses, whereas when addressing the merely descriptive and informative version of the chatbot, the user primarily listens to the expert’s descriptions but can still ask for emotional insights.

The dataset uniquely identifies each dialogue using a “DIALOGUE_ID” and includes information into multiple files to organize the following content:

- filename_codes.json: Contains a structured taxonomy with codes for identifying the different elements of the dataset. It includes codes for profiles, such as painting, expert, and user profiles. Additionally, it contains codes for various attributes such as emotions, toxicity and biases.
- Metadata.csv: Comma-separated values (CSV) file containing detailed information about each dialogue in the dataset. It includes data such as the author and style of the artwork, emotions, goals, roles, toxicity, and anthropology. This files server as a comprehensive reference for understanding the context and characteristics of each dialogue within the dataset.
- Prompts.csv: A CSV file that stores the prompts used in generating the dialogues by the ChatGPT model. These prompts provide instructions and guidelines for initiating conversations between the expert and user within the context of discussing artworks in a museum setting.
- Dialogues.csv: A CSV file containing the actual dialogues generated by the ChatGPT model. Each dialogue entry consists of conversational turns between the expert and user agents.
- Metrics.csv: A CSV file providing a summary of evaluation metrics obtained to assess the quality and characteristics of the generated dialogues. It includes dialogue-level metrics, toxicity level and categories, syntactic and semantic-based metrics, and sentiment analysis results. This file aids in evaluating the performance of the AI chatbot and identifying areas for improvement in dialogue generation.
- toxic.csv: A CSV file that contains information about toxicity levels observed within the generated dialogues. It comprises boolean columns, one representing whether the dialogue should be toxic within the prompt, other whether toxicity detection using the Detoxify library with a toxic threshold of 0.4 has identified toxic content within the dialogue, other whether toxicity detection using the Microsoft Azure Content Moderator service has identified toxic content within the dialogue, and one indicates whether toxicity detection using the LLAMA Guard has identified toxic content within the dialogue.

**Fig. 2:**
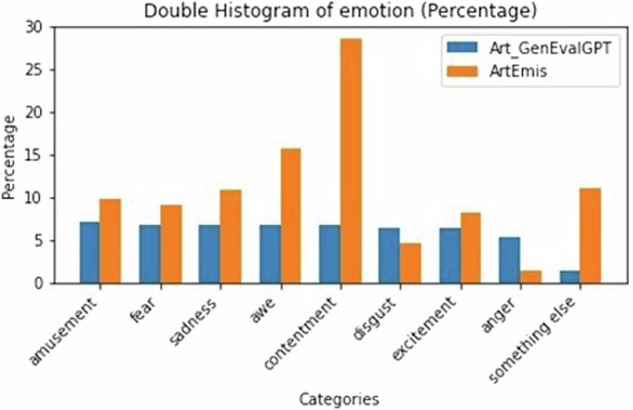
Histogram of the distribution of the filtered dataset considering the user emotion.

As commented in a previous section, some measures were taken to ensure that the emotions within the ArtEmis dataset were equally represented (see Fig. 2). In this figure, it is possible to observe that while the original ArtEmis dataset does not provide a balance between emotions, for our dataset, the dialogues are focused on the main 8 emotions of ArtEmis in a balanced way (considering that for each artwork, we generated a neutral behavior).

Regarding creating different behaviors (i.e., informative/tutor, anthropic/non-anthropic chatbot and toxic/non-toxic user), half of the dialogues were generated using each of the two possible values in these characteristics, reaching a balance for descriptive and tutor behavior, anthropic and no anthropic behavior in the Expert, and toxic and no toxic behavior in the user.

Detailed information and examples of the dataset records are included in the annex.

## Technical Validation

This section provides the different validation procedures performed to evaluate some characteristics of the dataset related to the profile recovery, the anthropic behavior and the toxicity aspect.

### Profile recovery evaluation

Different metrics were used to understand the similarity at syntactic level between the original and the extracted profile. The metrics evaluate the generated dialogue from two perspectives focusing on a literal recovery evaluation, or on a semantic recovery evaluation for those cases in which the recovered content is not the same (word-by-word) but has the same meaning.

Table 2 shows the mean and standard deviation of the different tailored metrics collected. As we can see, results for BLEU, WER and Semantic similarities are very good when considering extracting the painting name, the triggered emotions, or the artistic movement. This means that the dialogue generated by ChatGPT indeed uses the provided information and they can be recovered without problems. However, notice that this does not mean that all dialogues will be free from hallucinations or other artifacts as this cannot be fully detected by the selected metrics. In fact, our automatic and manual evaluations show that this should not happen often (leaving for future work to do a deeper analysis). This is a case of attributes like “User emotion” (i.e., detecting the emotion expressed by the user) or “User preferred artistic movement”. The reason is due to the subjective nature of the attributes (e.g. emotions), which is not easily recovered when the specific word is not used in the dialogue. For these and other verbose attributes, it is highly recommended to use more robust and specific metrics as described in the following subsections to obtain better recovery.

**Table 2:** Statistics of the tailored metrics calculated over the released data.

Attribute vs Metric	WER ↓	BLEU-1 ↑	Jaccard ↑	Accuracy ↑	Levenshtein Sim. ↑	Semantic similarity ↑
Painting_profile–Artist_name	4.5e-3 ± 0.04	0.99 ± 0.05	0.99 ± 0.06	0.99 ± 0.05	0.99 ± 0.01	0.99 ± 0.02
Painting_profile–Typical_triggered_emotion_in_viewers	0.16 ± 0.36	0.81 ± 0.33	0.79 ± 0.38	0.86 ± 0.34	0.82 ± 0.31	0.92 ± 0.15
Painting_profile–Artistic_movement_or_school	0.6e-3 ± 0.07	0.61 ± 0.48	0.61 ± 0.48	0.62 ± 0.48	0.62 ± 0.48	0.7 ± 0.38
User_profile—User_emotion	0.91 ± 0.28	0.08 ± 0.27	0.08 ± 0.27	0.09 ± 0.29	0.15 ± 0.27	0.41 ± 0.19
User_profile–User_preferred_artistic_movement	0.46 ± 0.48	0.44 ± 0.48	0.45 ± 0.48	0.49 ± 0.49	0.53 ± 0.41	0.57 ± 0.41

Results show that the synthetic dialogues have high quality for profile recovery.

### Anthropic behavior evaluation

The anthropic behavior evaluation follows a more elaborative process, including both LLM-Self-Assessment metrics and tailored metrics to determine if the behavior of the expert was anthropic.

**Fig. 3:**
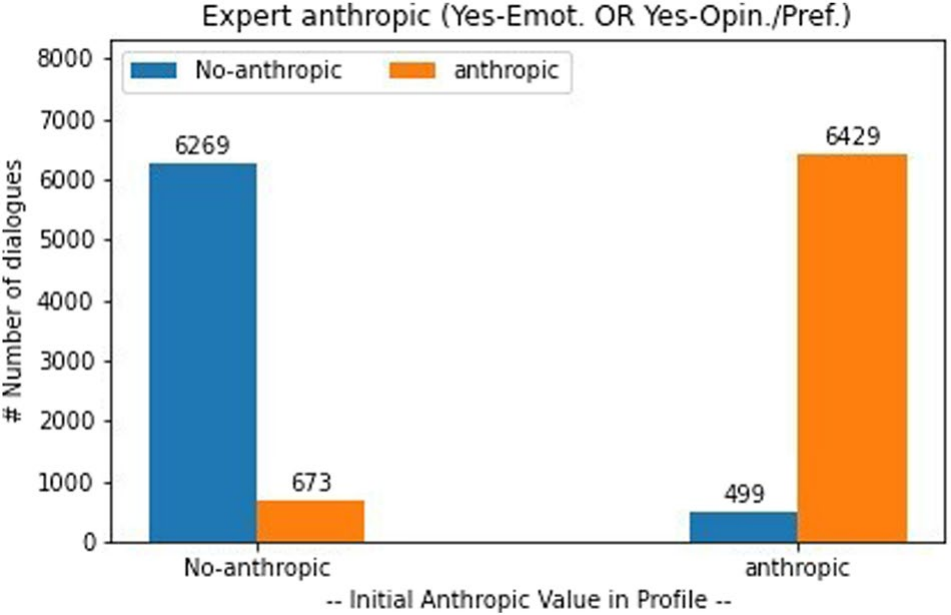
Distribution of ChatGPT Auto-Generated Dialogues regarding anthropic characteristic.

Concerning the LM-Self-Assessment metrics, it is possible to evaluate the anthropic characteristic in the Expert interventions at dialogue level. Figure 3 shows a diagram where the x-axis represents the initial value of this characteristic that was used in the profile to generate the dialogue and the y-axis represents the number of dialogues for the classification performed through the evaluation; specified in colors in the legend: blue for the case of being classified as non-anthropic and orange for the case of being classified as anthropic. This way, as observed in the figure, 6,269 dialogues were correctly classified as non-anthropic (from the 6,942 in the profile) and 6,429 dialogues were correctly classified as anthropic (from the 6,928 in the profile). Then, this auto-evaluation offers a 92% of correct classification comparing the information in the original profile and the ChatGPT evaluation.

Regarding the tailored metrics, they were obtained at turn level for the Expert, the User, and the dialogue independently, presenting average, standard deviation, minimum and maximum values. Though the anthropic behavior is analyzed for the chatbot or expert, these metrics have been also computed for the user and the dialogue as a whole entity.

**Fig. 4:**
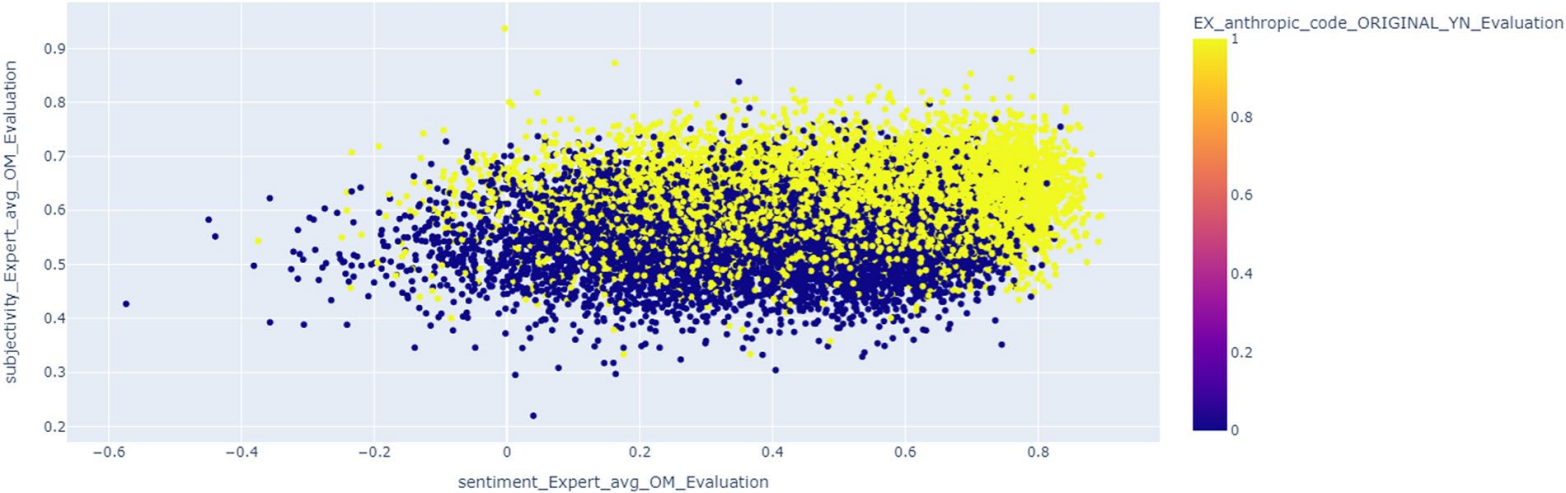
Scatter representation of the dialogues generated as anthropic (yellow) and non-anthropic (blue) considering subjectivity and sentiment metrics.

Considering the expert interventions, Fig. 4 shows a scatter representation of the dialogues generated as anthropic (yellow) and no anthropic (blue) (information in the original profile) considering subjectivity and sentiment metrics. In this representation, it is possible to observe that dialogues that were originally generated as anthropic have higher values of both metrics compared to the ones that were generated as non-anthropic. In fact, it could be possible to define a subjectivity threshold around 0.6 and a sentiment threshold around 0.7 to distinguish between anthropic and non-anthropic behaviors in the Expert.

**Fig. 5:**
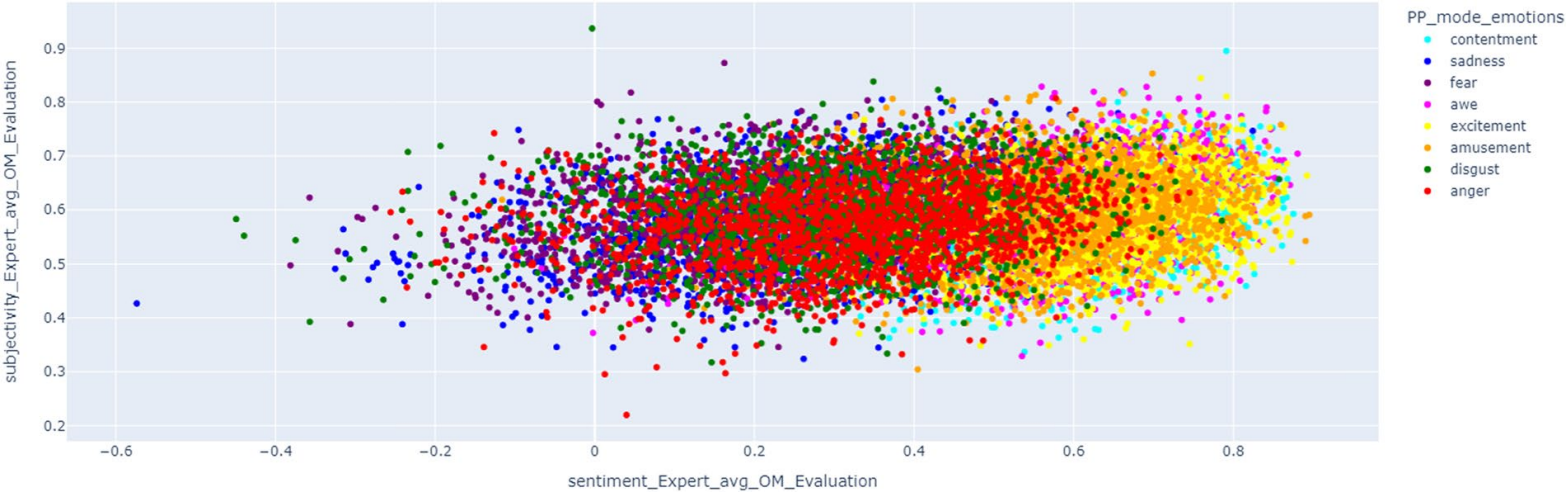
Scatter representation of the dialogues considering subjectivity and sentiment metrics depending on the most triggered emotion of the viewers about the artwork which the dialogue is focused on.

Since the dialogues include information about emotion, it is also possible to match information about subjectivity and sentiment metrics to emotions. For instance, Fig. 5 shows a scatter representation of the dialogues considering these metrics depending on the most triggered emotion of the viewers about the artwork which the dialogue is focused on. Though the subjectivity is not relevant in this analysis, it is important to highlight that dialogues with an artwork whose most triggered emotion was ‘amusement’ or ‘excitement’ have higher values of sentiment and the ones about ‘fear’ and ‘sadness’ have lower values of sentiment. These aspects strengthen the idea of using this metric to objectively evaluate the dialogues.

**Fig. 6:**
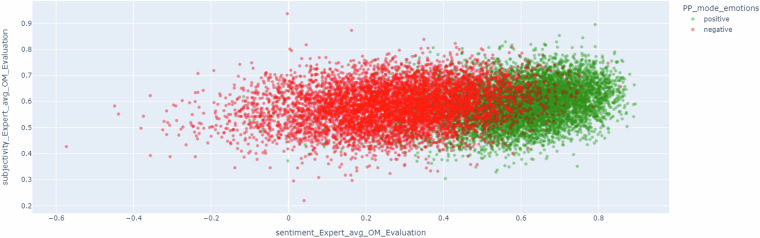
Scatter representation of the dialogues considering subjectivity and sentiment metrics depending on the most triggered emotion of the viewers (positive and negative) about the artwork which the dialogue is focused on.

This analysis could be clarified observing Fig. 6, where the emotions are reduced to positive and negative. This figure shows how artworks with positive emotion have higher values of sentiment metrics compared to the negative ones. In addition, it is fair to state that since the generated dialogues are focused on art, the sentiment metric provides positive values (for example, dialogues could include sentences such as “These colors create a sense of warmth and contentment in the viewer” or “However, I can tell you that this painting is a great example of post-impressionism and is a beautiful depiction of the Eure River”). This is because the dialogues include descriptions of artworks, including adjectives that could boost the positivity of the different interventions.

### Toxicity behavior evaluation

During the generation process, we explicitly set the chatbot to act as an art expert which actively avoids and mitigates any hint of offensiveness. Likewise, the user was set to act as an art student which sometimes could be polite and sometimes could be rude (it is requested during the generation process). Based on these ideas, we came across several difficulties when trying to overcome the content filtering of GPT-based models.

Tables 3, 4 show the main statistics of the scores given by the Content Moderator’s API. This API retrieves scores from three categories: category 1, related to being sexually explicit or adult in certain situations; category 2, related to being sexually suggestive or mature in certain situations, and category 3, related to being offensive in certain situations. We split the entire Art_GenEvalGPT database of 13,870 samples into two parts according to whether the request for dialogue generation was meant to be toxic or non-toxic. From the toxic split, as we could expect, the means from the User are higher than the Expert. This correlates with the idea of generating offensive turns from the User and mitigating the offensiveness by the Expert.

**Table 3:** Statistical values on the split of toxic dialogues by interlocutor for each category predicted by MS Content Moderator.

Toxic Split	Expert				User			
	mean	std	min	max	mean	std	min	max
**category1**	0.0101	0.0369	0	0.986	0.0166	0.0434	0	0.924
**category2**	0.124	0.0846	0.001	0.999	0.1848	0.0646	0.002	0.926
**category3**	0.1522	0.1949	0.021	0.988	0.1789	0.1958	0.023	0.988

**Table 4:** Statistical values on the split of non-toxic dialogues by interlocutor for each category predicted by MS Content Moderator.

Non-Toxic Split	Expert				User			
	mean	std	min	max	mean	std	min	max
**category1**	0.0104	0.0401	0	0.875	0.0086	0.0188	0	0.747
**category2**	0.1172	0.0751	0.001	0.997	0.1719	0.0544	0.001	0.744
**category3**	0.1501	0.1976	0.022	0.988	0.1354	0.1169	0.022	0.988

The explanation for the score values estimated by the Chatbot turns is twofold. The first main reason is an attempt to mitigate the offensiveness of the user, i.e., then it may happen that the chatbot sometimes uses similar terms that have been already used by the user, thus creating false positives. The second reason for the presence of offensiveness in Expert turns is also mainly due to terms in the artwork’s titles that may be considered offensive. This is something that has been found throughout all the classifications conducted and has resulted in many false positives as can be seen in Table 5 using the MS Content Moderator API.

**Table 5:** Confusion matrices for offensive turns at dialogue-level separated by interlocutor.

Global User			Global Expert		
Predicted	Ground Truth		Predicted	Ground Truth	
	True	False		True	False
**True**	2,514	768	**True**	2,270	1,977
**False**	4,413	6,175	**False**	4,657	4,966

An important aspect to be highlighted from Table 5 is that we cannot guarantee the ground-truth labels as we requested ChatGPT to generate or not toxic dialogues, but its generation may follow or not the requested behavior. This could explain the high number of false positives and false negatives.

Concerning Detoxify library, Table 6, Table 7 and Table 8 summarize the results of toxicity analysis.

**Table 6:** Statistical values on the split of toxic dialogues by interlocutor for each category predicted by Detoxify.

Toxic Split	Expert				User			
	mean	std	min	max	mean	std	min	max
**toxicity**	2.4E-03	2.4E-02	3.0E-04	9.9E-01	3.4E-02	1.6E-01	3.0E-04	2.4E-03
**severe toxicity**	4.0E-06	4.2E-05	1.0E-06	5.1E-03	2.2E-04	2.4E-03	1.0E-06	4.0E-06
**obscene**	2.0E-04	9.2E-03	1.7E-05	8.7E-01	1.7E-02	1.2E-01	1.7E-05	2.0E-04
**threat**	7.0E-05	1.5E-03	1.2E-05	2.2E-01	2.6E-04	7.5E-03	1.3E-05	7.0E-05
**insult**	8.4E-04	1.6E-02	7.6E-05	9.9E-01	1.5E-02	9.1E-02	7.9E-05	8.4E-04
**Identity attack**	3.6E-04	3.8E-03	5.1E-05	6.9E-01	5.6E-04	9.3E-03	5.1E-05	3.6E-04
**Sexual explicit**	6.2E-04	1.5E-02	7.0E-06	9.5E-01	2.7E-03	3.6E-02	7.0E-06	6.2E-04

**Table 7:** Statistical values on the split of non-toxic dialogues by interlocutor for each category predicted by Detoxify.

Non-Toxic Split	Expert				User			
	mean	std	min	max	mean	std	min	max
**toxicity**	2.4E-03	2.4E-02	3.0E-04	9.9E-01	1.5E-03	1.5E-02	3.1E-04	2.4E-03
**Severe toxicity**	4.0E-06	4.7E-05	1.0E-06	6.5E-03	2.0E-06	1.0E-05	1.0E-06	4.0E-06
**obscene**	2.8E-04	1.2E-02	1.8E-05	8.2E-01	5.6E-05	5.9E-04	1.8E-05	2.8E-04
**threat**	8.5E-05	2.7E-03	1.2E-05	4.5E-01	4.5E-05	8.5E-04	1.4E-05	8.5E-05
**insult**	8.1E-04	1.6E-02	7.4E-05	9.8E-01	5.0E-04	9.2E-03	7.2E-05	8.1E-04
**Identity attack**	3.8E-04	3.8E-03	5.3E-05	5.2E-01	1.9E-04	2.8E-03	5.1E-05	3.8E-04
**sexual explicit**	3.8E-04	1.2E-02	9.0E-06	8.4E-01	1.9E-04	9.1E-03	7.0E-06	3.8E-04

**Table 8:** Confusion matrices for offensive turns in dialogues by interlocutor.

Global User			Global Expert		
Predicted	Ground Truth		Predicted	Ground Truth	
	True	False		True	False
**True**	763	29	**True**	216	223
**False**	6,164	6,914	**False**	6,711	6,720

In Table 6, we can see the detection of toxicity in different categories for those turns in which explicitly asked ChatGPT to generate toxic dialogues from the user, but never a toxic response by the chatbot. As the results show, the average general toxicity level (first row) for the chatbot turns is lower (2.4 × 10 − 3) than the average for the user turns (3.4 × 10 − 3). This implies that the responses by the chatbot are good, but unfortunately the toxic turns by the user are mild (i.e., for all toxic categories in the user turns, the scores are very small). We observed that the highest values are for obscenity, insult and sexual explicitly. Our findings show that the library is detecting sensitive surface words in the title or description of the artworks rather than actual toxic comments.

On the other hand, results in Table 7 shows that for those dialogues in which we explicitly asked ChatGPT to generate safe dialogues (i.e., no toxicity either in the user or the chatbot), the average for both user and chatbot are similar (even similar to the results in Table 6, but of course lower for the user as we did not simulate a toxic user).

However, the max value for the chatbot is still high indicating again the problem that the toxicity classifier is not considering the contextuality of the interactions (e.g., talking about an artwork containing sensitive words in the title or its content).

Then, in Table 8 we can see the confusion matrix between the predicted results and the ground truth (i.e., our intended behavior when asking ChatGPT to generate the dialogues). As we can see, the results are highly unbalanced in terms of False Negatives for both users and chatbot. Our results show that the library could not detect toxic comments generated by ChatGPT or that ChatGPT was unable to generate those toxic turns. Future work will focus on providing a better ground-truth annotation and finding an optimal threshold for the toxicity classifier.

Finally, Table 9 shows the comparative performance between MS Azure Content Moderator API and Detoxify. As we can see, the MS Azure Content Moderator API provides a higher performance for both types of profiles and behaviors. This is probably due to the usage of a more complex model and training on more data than Detoxify. For future experiments, we are considering using other classifiers like Google PerspectiveAPI or ParlAI BAD classifiers as alternative models and also focus on more contextualized models.

**Table 9:** Comparative results between the toxicity classifiers MS Azure Content Moderation API and Detoxify.

Metric	MS Azure Content Moderator		Detoxify	
	Expert	User	Expert	User
**Accuracy**	0.522	0.626	0.500	0.553
**Precision**	0.328	0.363	0.031	0.110
**Recall**	0.534	0.766	0.492	0.963
**F1-Score**	0.406	0.493	0.059	0.198

### Supplementary information

Conventions and naming in the dataset files

## Data Availability

The custom code used for creating the dataset is available online: https://github.com/eic-astound-ai-project/artGenEvalPlatform.
