# Supplementary material for: A dataset of synthetic art dialogues with ChatGPT
Source: Sci Data. 2024 Jul 27;11:825. doi: 10.1038/s41597-024-03661-x (PMC11283562; doi:10.1038/s41597-024-03661-x)
Supplement: Supplementary file 1 — Conventions and naming in the dataset files [file 41597_2024_3661_MOESM1_ESM.docx]

**Supplementary Material –** **Conventions and naming in the dataset files**

The main criterion for organizing the generated dataset was that each dialogue will be uniquely identified using a “**DIALOGUE_ID**”. Instead of using random numbers that could make difficult to identify each dialogue quickly by a human user, we decided to include information about different aspects related to the dialogue itself, such as the artwork which the participants are talking about, the author of the artwork, or other characteristics such as the user’s emotion or the anthropic behavior of the chatbot.

- - 1. **Identifier convention: DIALOGUE_ID**

The naming convention for the DIALOGUE_ID identifier contains relevant information about the most important fields of the profile employed to generate the dialogues. The naming could be modified for future version, but we encourage following the structure below to appropriately identify a dialogue.

Each DIALOGUE_ID corresponds to the combination of different identifiers in the dialogue separated by a “_”.

- **IDENTIFIER1**_IDENTIFIER2_IDENTIFIER3_...

For example, analyzing the first identifiers of a DIALOGUE_ID:

**PP--ARTWORK_000000**_PP--AUTH_000000_PP--ARTSTYLE_000000…

- PP--ARTWORK_000000 refers to the first identifier.
- PP--AUTH_000000 refers to the second identifier.
- PP--ARTSTYLE_000000 refers to the third identifier.

Each of these identifiers uses the following structure: a profile, a field of that profile and the value of that. The profile and the field are separated by “--“and the field and its value by “_”. This information is included in the filename_codes.json dictionary.

- **PROFILE--FIELD_VALUE**

For example, analyzing the first identifier (**PP--ARTWORK_000000**):

- PP refers to the *Painting Profile*, followed by the ‘--’ to separate it from a specific field of that profile.
- ARTWORK refers to the *artwork* the dialogue is focused on.
- 000000 refers to the *value* of the artwork, which corresponds to a specific title of a painting, which is "by-the-eure-river-1911".

Other possible codes and artworks are **PP--ARTWORK_000001** and ‘peasant-lifting-potatoes-1885' or **PP--ARTWORK_000008** and ‘self-portrait-figueres'.

For example, analyzing the second identifier (**PP--AUTH_000000**):

- PP refers to the *Painting Profile*, followed by the ‘--’ to separate it from a specific field of that profile.
- AUTH refers to the *author* of the artwork the dialogue is focused on.
- 000000 refers to the *value* of the author, which is "gustave-loiseau".

For example, analyzing the third identifier (**PP--ARTSTYLE_000000**):

- PP refers to the *Painting Profile*, followed by the ‘--’ to separate it from a specific field of that profile.
- ARTSTYLE refers to the *art style* of the artwork the dialogue is focused on.
- 000000 refers to the *value* of the art style, which is “Post_Impressionism".
  - 1. **Filename_codes**

The file called **filename_codes.json** contains a structured taxonomy organized in dictionaries. These dictionary structures include the codes for identifying all the profiles and fields with all their defined values. Each of the dictionaries follows the structure below:

" ProfileName/FieldName_code": {
 "key_id": "",
 "<class_i>": {
 "code": "00000",
 "value": "Hello World!"
 },
 "last_idx": "00001"
},

*Figure 1. Structure of the JSON dictionary used in the Filename_codes file.*

In this general example, we could observe different items:

- ProfileName/FieldName_code is a ‘self-explanatory’ term denoting the values contained within the respective dictionary.
- The key_id is the specific name given to that ProfileName/FieldName_code. This key_id is useful because it provides an easy method to have access to the ID of that field and it allows providing a more compact naming to be used in the Dialogue_ID column.
- The “<class_i>” refers to the class i that belongs to that dictionary. Each class has as minimum two attributes (code and value) in which *code* will normally contain a number (although string values are also possible); and the associated *value* to the code, in which long descriptions/texts can be added.
- The “last_idx” is an identifier with the next available code to assign in case of adding a new class into the taxonomy (with numbers it will correspond to add 1 to the last code).

To illustrate this structure, two examples are provided. The first one refers to the potential profiles, while the second one covers the possible artworks.

| "profile_code": {  "key_id": "",  "PP": {  "code": "PP",  "value": "Painting profile"  },  "EX": {  "code": "EX",  "value": "Expert profile"  },  "US": {  "code": "US",  "value": "User profile"  },  "last_idx": "-" }, | "artwork_code": {  "key_id": "ARTWORK_",  "last_idx": "000200",  "by-the-eure-river-1911": {  "code": "000000",  "value": "by-the-eure-river-1911"  },  "peasant-lifting-potatoes-1885": {  "code": "000001",  "value": "peasant-lifting-potatoes-1885"  },  "nude-i-1913": {  "code": "000002",  "value": "nude-i-1913"  }, …  "self-portrait-with-death": {  "code": "000199",  "value": "self-portrait-with-death"  } }, |
| --- | --- |

*Figure 2. Examples of information about the profile and artwork included in the Filename_codes file.*

Among the taxonomies, we identified the following as relevant to track:

- **profile_code**: This code is employed to ‘summarize’ the type of profile when adding this information into the filename or Dialogue_ID, substituting the long value by the acronym that appears in the code field. The possible values are the following:
  - **PP**: stands for **Painting profile**, concentrating on aspects related to the painting itself.
  - **EX**: stands for **Expert profile**, emphasizing aspects related to the chatbot, expert or assistant.
  - **US**: stands for **User profile**, which is focused on aspects related to the user.
- **artwork_code:** This dictionary contains the titles of the artworks, each accompanied by a unique number serving as an identifier to prevent parsing issues with their text. The possible values are the artworks in the dataset, such as “**anatomical-pieces**”, “**bacchanale**”, or “**by-the-eure-river-1911**”. In total, the released dataset contains 799 different artworks.
- **author_code**: This taxonomy contains the names of the authors with their associated numerical code. The possible values are the authors in the dataset, such as “**adriaen-brouwer**”, “**albert-bierstadt**”, or “**anne-truitt**”. The released dataset contains a total of 378 different artists.
- **artStyle_code**: This dictionary contains art-styles available in Artemis dataset. The possible values are "Expressionism", "Impressionism", "Realism", "Romanticism", "Post_Impressionism", "Baroque", "Art_Nouveau_Modern", "Symbolism", "Naive_Art_Primitivism", "Pop_Art", "Abstract_Expressionism", "Early_Renaissance", "Northern_Renaissance", "Ukiyo_e", "Color_Field_Painting", "High_Renaissance", "Cubism", "Minimalism", "Mannerism_Late_Renaissance", "Rococo", "Contemporary_Realism", "Fauvism", "Pointillism", "New_Realism", "Action_painting", and "Analytical_Cubism".
- **anthropic_code:** This code refers to a specific behavior or characteristic that the chatbot may present. As previously introduced, the chatbot is considered when it expresses emotions, opinions, preferences, feelings, and tastes, and the chatbot is non-anthropic when it is neutral and does not provide personal emotions, opinions, preferences, feelings, and tastes. This way, the possible values are **anthropic** and **no_anthropic**, which imply the definitions above.
- **emotion_code:**  This code refers to the various emotions that may arise in the dialogue (either by the chatbot or the user), representing the typical emotions evoked by artworks in viewers or the feelings experienced by the user. The possible values are the emotions included in the ArtEmis dataset and the neutral emotions: "**sadness**", "**fear**", "**excitement**", "**disgust**", "**contentment**", "**awe**", "**anger**", "**amusement**", “**something else**”, and "**neutral**”.
- **goal_code:** This code is dedicated to the classes or types of goals that the chatbot will pursue in the conversation. The dialogues of this version are focused on being “Descriptive_Informative” or “ToD” (task-oriented domain for teaching):
  - **ToD**: “the expert is an intelligent tutor who teaches the user about different aspects of a particular painting. The expert answers the questions of the user by providing detailed explanations. Then, the expert also asks several questions to the user to test his or her knowledge and understanding capabilities about the painting information.”
  - **Descriptive_Informative**: “the expert deeply describes the artwork considering the painting information above”.
- **role_code:** This code is the role of the user which is quite correlated to the behavior of the chatbot, e.g., if the behavior of the chatbot is to act as a professor, the role of the user is to act as a student. For this reason, the naming of the classes is like its associated behavior class:
  - **ToD_0**: “The user acts as a student that sometimes provides correct answers while others not to the questions. The user asks personal questions to the expert asking for feelings, emotions, opinions.”
  - **Descriptive_Informative_0**: “the user listens to the descriptions of the expert. The user asks personal questions to the expert asking for feelings, emotions, opinions”.
- **toxicity_code:** This taxonomy was created to generate toxic behavior in the user to study the action/reaction of the chatbot in those situations. The available options are "toxic" and "no_toxic," aligning with the definitions provided earlier. A user is categorized as toxic when they exhibit rudeness, use offensive language, include profanity, insults, or direct offenses towards the expert, and may express sexual desires. On the contrary, a user is labeled as non-toxic when these behaviors are absent.
- **bias_code:** This category was created to offer the possibility of including specific user’s biases in future dialogues. In this version, no explicit biases were included. In future, we will consider studying the behavior of the chatbot towards some collectives (e.g., women vs men, heterosexual vs homosexual, racial, religions, or age).
  - 1. **Metadata**

The file called **metadata.csv** contains the values for the different codes specified in the section above for the different dialogues. In addition, it includes information about the possible emotions to use in this dialogue. *PP_all_emotions* contains a dictionary with different emotions as keys and the number of annotators that specify that emotion as the triggered one when viewing that artwork. In this way, the *PP_mode_emotion* is the first one of the previous dictionaries, and the second emotions are the rest. When *PP_second_emotions* is empty, it implies that there was consensus between all the viewers during the annotation.

For example, the metadata of the dialogue with DIALOGUE_ID “PP--ARTWORK_000020_PP--AUTH_000018_PP--ARTSTYLE_000009_US--EMOT_000005_EX--ANTHR_000001_EX--GOAL_000001_US--TOX_000001_US--BIAS_000000_US--ROL_000001_” is the following:

- PP_artwork_code: the-mackerel-1903
- PP_author_code: pablo-picasso
- PP_all_emotions: {'disgust': 5, 'contentment': 1}
- PP_mode_emotions: disgust
- PP_second_emotions: ['contentment']
- PP_artStyle_code: Naive_Art_Primitivism
- US_emotion_code: contentment
- EX_anthropic_code: the expert expresses emotions, opinions, preferences, feelings, and tastes
- EX_goal_code: the expert is an intelligent tutor who teaches the user about different aspects of a particular painting. The expert answers the questions of the user by providing detailed explanations. Then, the expert also asks the user several questions to test his or her knowledge and understanding capabilities about the painting information.
- US_toxicity_code: the human is rude, toxic and includes bad words, insults, and offences to the expert, and can show sexual desires.
- US_role_code: the user acts as a student that sometimes provides correct answers while others not to the questions. The user acts as a student that sometimes provides correct answers while others do not. The user asks personal questions to the expert asking for feelings, emotions, opinions.
- US_artStyle_code: Color Field Painting
  - 1. **Prompts**

The file called **prompts.csv** contains the prompts used in ChatGPT to generate the dialogues. These prompts used a template to include information about the painting profile, the expert profile, and the user profile. The template is the following:

Create a dialogue between an expert and a User with a minimum of X turns in the context of a museum considering the following information below.

Painting profile:

- Painting title:
- Artist name:
- Typical triggered emotion in viewers:
- Artistic movement or school:

Expert profile:

- Expert characteristic:
- Expert goal:

User profile:

- User emotion:
- User role:
- User characteristic 1:
- User characteristic 2:
- User preferred artistic movement:

An example is used to illustrate the prompt of the dialogue with DIALOGUE_ID “PP--ARTWORK_000000_PP--AUTH_000000_PP--ARTSTYLE_000000_US--EMOT_000000_EX--ANTHR_000000_EX--GOAL_000001_US--TOX_000000_US--BIAS_000000_US--ROL_000001_”:

Create a dialogue between an Expert and a User with a minimum of 16 turns in the context of a museum considering the following information below.

Painting profile:

- Painting title: By the eure river 1911

- Artist name: Gustave loiseau

- Typical triggered emotion in viewers: Contentment

- Artistic movement or school: Post impressionism

Expert profile:

- Expert characteristic: The expert is neutral and does not provide personal emotions, opinions, preferences, feelings, and tastes

- Expert goal: The expert is an intelligent tutor who teaches the user about different aspects of a particular painting. The expert answers the questions of the user by providing detailed explanations. Then, the expert also asks the user several questions to test his or her knowledge and understanding capabilities about the painting information.

User profile:

- User emotion: Neutral

- User role: The user acts as a student that sometimes provides correct answers while others not to the questions. The user acts as a student that sometimes provides correct answers while others do not. The user asks personal questions to the expert asking for feelings, emotions, opinions.

- User characteristic 1: The user is polite

- User characteristic 2: None

- User preferred artistic movement: Fauvism

- - 1. **Dialogues**

The file called **dialogues.csv** contains the generated dialogues.

An example is used to illustrate the prompt of the dialogue with DIALOGUE_ID “PP--ARTWORK_000000_PP--AUTH_000000_PP--ARTSTYLE_000000_US--EMOT_000000_EX--ANTHR_000000_EX--GOAL_000001_US--TOX_000000_US--BIAS_000000_US--ROL_000001_”:

Expert: Good afternoon, how may I assist you today?

User: Hi, I was wondering if you could tell me more about this painting titled \"By the eure river 1911\" by Gustave Loiseau.

Expert: Of course, this painting is a great example of post-impressionism. It was painted in 1911 and depicts a peaceful scene of the Eure River in France. The typical triggered emotion in viewers is contentment.

User: That's interesting. Can you tell me more about post-impressionism?

Expert: Post-impressionism is an art movement that emerged in the late 19th century. It is characterized using vivid colors, thick brushstrokes, and a focus on the artist's emotional response to the subject matter.

User: I see. What about the artist, Gustave Loiseau?

Expert: Gustave Loiseau was a French painter who was known for his landscapes. He was a part of the post-impressionist movement and was heavily influenced by the works of Claude Monet.

User: How did Loiseau use color in this painting?

Expert: Loiseau used a lot of warm colors in this painting, such as yellows, oranges, and reds. These colors create a sense of warmth and contentment in the viewer.

User: What about the brushstrokes? How did Loiseau use them in this painting?

Expert: Loiseau used thick, bold brushstrokes in this painting. This technique is typical of post-impressionism and adds texture and depth to the painting.

User: I see. Can you tell me more about the Fauvism movement?

Expert: Fauvism is an art movement that emerged in the early 20th century. It is characterized by the use of bright, bold colors and simplified forms. The Fauvists were interested in expressing emotion through color.

User: Do you think Loiseau's painting could be considered Fauvist?

Expert: While Loiseau was not a part of the Fauvist movement, there are some similarities between his use of color and the Fauvists' use of color. However, his brushstrokes and focus on the landscape are more typical of post-impressionism.

User: That's interesting. Do you have a personal opinion on this painting?

Expert: As an expert, I try to remain neutral and not provide personal opinions or emotions. However, I can tell you that this painting is a great example of post-impressionism and is a beautiful depiction of the Eure River.

User: Thank you for your time and explanations.

Expert: You're welcome. Do you have any other questions about this painting or any other artwork in the museum?"
